# Money and transmission of bacteria

**DOI:** 10.1186/2047-2994-2-22

**Published:** 2013-08-28

**Authors:** Habip Gedik, Timothy A Voss, Andreas Voss

**Affiliations:** 1 Department of Infectious Diseases and Clinical Microbiology, Ministry of Health Okmeydanı Training and Research Hospital, Istanbul, Turkey; 2 SGN, Nijmegen, The Netherlands; 3 Department of Medical Microbiology and Infection Control, Canisius-Wilhelmina Hospital and Radboud University Nijmegen Medical Centre, P.O. Box 9015, 6500 GS Nijmegen, The Netherlands

## Abstract

Money is one of the most frequently passed items in the world. The aim of this study was to ascertain the survival status of bacteria including *Staphylococcus aureus*, *Escherichia coli*, and Vancomycin- Resistant Enterococci (VRE) on banknotes from different countries and the transmission of bacteria to people who come in contact with the banknotes. The survival rate was highest for the Romanian Leu yielding all three microorganisms used after both three and six hours of drying. Furthermore, the Leu was the only banknote to yield VRE after one day of drying. Other currencies either enabled the survival of Extended-Spectrum Beta-Lactamases (ESBL) and VRE (e.g. Euro), but not of MRSA, or the other way round (e.g. US Dollar). While a variety of factors such as community hygiene levels, people’s behaviour, and antimicrobial resistance rates at community level obviously have influence on the transmission of resistant microorganisms, the type of banknote-paper may be an additional variable to consider.

## Introduction

Globally, money is one of the items most frequently passed from hand to hand. During its passing, money can get contaminated and may thus plays a role in the transmission of microorganisms to other people. For example, money may get contaminated with microorganisms from the respiratory- and gastro-intestinal tract during counting. Money is not usually suitable for the survival of microorganisms, except for some that are resistant to external conditions and non-resistant forms of spores [1,2]. In addition, the general hygiene levels of a community or society may contribute to the amount of microbes found on coins and notes, and thus the chance of transmission during handling of money. While antimicrobial resistance has steadily been increasing, e.g. with Extended-Spectrum Beta Lactamases (ESBL) producing *Escherichia coli* and *Klebsiella spp*[3], contaminated banknotes and coins contribute to the transmission of these multi-drug resistant microorganisms in the community.

While the kind of isolated bacteria between studies can vary, due to the methods used, season, environmental conditions, sort of money (coin or banknote) or local community flora, in general, Gram positive bacteria were the most predominant [4-9].

The aim of this study was to ascertain the survival status of bacteria including *Staphylococcus aureus*, *E. coli*, and Vancomycin-Resistant Enterococci (VRE) on banknotes from different countries and the transmission of bacteria to volunteers getting in contact with contaminated banknotes under experimental conditions.

## Material and method

This study was conducted in the medical microbiology laboratory of Canisius Wilhelmina Ziekenhuis in 2012. The first step of study was to inoculate a few colonies of methicillin-resistant *S.aureus (*MRSA) ATCC 43300, Vancomycin-resistant *Enterococcus feaecium (*VRE) ATCC 51559 and an extended spectrum of beta lactamases (ESBL) producing *E. coli* ATCC 25922 strains into 5 ml Tryptic Soy broth medium in tubes, which then were incubated at 35°C for 24-h. All banknotes were inoculated with 8 dilutions of a serial dilution of the original inoculum, by spreading 100 μl from each tube onto different described areas of the banknotes which were previously sterilized under ultraviolet light radiation. As a control, 100 μl of all dilutions were inoculated onto Columbia agar. After a 24-h incubation at 35°C, all colony-forming units were counted, and the number of bacteria given onto the banknotes calculated. All experiments were done in duplicate. All banknotes were dried in ambient conditions. Cultures from the banknotes were taken by a moistened swab (0.9% saline) after 3-h, 6-h and 24-h. Swabs were directly inoculated onto Columbia agar and into tubes with 1 ml 0.9% saline. 100 μl from each tube were spread onto Columbia agar.

For the second part of the study, methicillin sensitive *S.aureus (*MSSA) ATCC 25923 and non-extended spectrum beta lactamases producing *E. coli* ATCC 35218 strains were inoculated into 5 ml (Tryptic Soy broth), incubated at 35°C for 24-h, A 100 μl inoculum of MSSA was spread onto one side of a US Dollar and Romanian Leu (RON) banknote, that has previously been sterilized by ultraviolet light radiation. In the same way, *E. coli* was spread onto one side of a Euro and a Romanian Leu (RON) banknote. After the banknotes were dried for 30 minutes, three people whose hands were disinfected by alcohol-based hand rub, and washed with sterile 0.9% saline, rubbed the banknotes for 30 seconds, respectively. After rubbing, the fingers were sampled by placing the fingertips directly on the 5% sheep-blood agar plate. Semi-quantitative colony counts were established after a 24-h incubation period for each person.

After the study was completed, all of the banknotes were sterilized by ultraviolet light radiation. The banknotes were not damaged during the study and brought into re-circulation.

## Results

Euro, US Dollar, Canadian Dollar, Croatian Luna, Romanian Leu (RON), Moroccan Dirham, and Indian Rupee banknotes were included into the first part of the study. Cultures of the Romanian Leu yielded all 3 multi-drug resistant pathogens; MRSA, VRE and ESBL-producing *E. coli*. The Canadian and (US) American Dollar only yielded MRSA; the Euro only ESBL-producing *E. coli*, the Indian Rupee only VRE, and the Croatian Luna did not yield any of the 3 microorganisms (Table 1). The Romanian Leu yielded all three microorganisms after both, three and six hours of drying, and it was the only currency which yielded a microorganism, VRE, after one day of drying. The Canadian Dollar yielded MRSA and VRE after six hours of drying. The Euro yielded ESBL-producing *E. coli* in the 3 and 6-h cultures, VRE in the 3-h culture, but at no time MRSA (Table 2).

**Table 1:** **Survival (semi-quantitative counts) of MRSA, VRE and ESBL-producing *E.coli *on different international banknotes**

**Country**	**Currency**	**MRSA**		**VRE**		**ESBL (+) *E.coli***	
		**Direct wet swab**	**Tube titration of swab**	**Direct wet swab**	**Tube titration of swab**	**Direct wet swab**	**Tube titration of swab**
**European countries**	**Euro**	Ø	Ø	+	Ø	+++	+++
**Croatia**	**Kuna**	Ø	Ø	Ø	Ø	Ø	Ø
**Romania**	**Leu (RON)**	+++	+++	+++	+++	+++	+++
**Morocco**	**Dirham**	Ø	Ø	+	Ø	++	Ø
**USA**	**Dollar**	+++	+++	++	Ø	+	Ø
**Canada**	**Canadian Dollar**	+++	+++	+	Ø	+++	Ø
**India**	**Rupee**	Ø	Ø	+	Ø	Ø	Ø

+++ = confluent growth, ++ = multiple segments, + = single colonies, Ø = no growth.

**Table 2:** **Survival (semi-quantitative counts) of MRSA, VRE and ESBL-producing *E.coli *on different international banknotes, over time**

**Time (h)**	**Currency**	**MRSA**	**VRE**	**ESBL (+) *E.coli***
**3**	**Euro**	**Ø**	**+**	**+**
	**Kuna**	**Ø**	**Ø**	**Ø**
	**Leu (RON)**	**+++**	**+++**	**+++**
	**Dirham**	**Ø**	**+**	**Ø**
	**Dollar**	**+**	**Ø**	**+**
	**Canadian Dollar**	**+**	**Ø**	**Ø**
	**Rupee**	**Ø**	**+**	**Ø**
**6**	**Euro**	**Ø**	**Ø**	**+**
	**Kuna**	**Ø**	**Ø**	**Ø**
	**Leu (RON)**	**+++**	**+++**	**+++**
	**Dirham**	**Ø**	**Ø**	**Ø**
	**Dollar**	**Ø**	**+**	**+**
	**Canadian Dollar**	**+**	**+**	**Ø**
	**Rupee**	**Ø**	**Ø**	**Ø**
**24**	**Euro**	**Ø**	**Ø**	**Ø**
	**Kuna**	**Ø**	**Ø**	**Ø**
	**Leu (RON)**	**++**	**Ø**	**Ø**
	**Dirham**	**Ø**	**Ø**	**Ø**
	**Dollar**	**Ø**	**Ø**	**Ø**
	**Canadian Dollar**	**Ø**	**Ø**	**Ø**
	**Rupee**	**Ø**	**Ø**	**Ø**

+++ = confluent growth, ++ = multiple segments, + = single colonies, Ø = no growth.

The second part of the study, the transmission experiments, was based on the results of the first part. Consequently, the Euro, Romanian Leu and US Dollar was used, after inoculation with *E. coli* (Euro) and MSSA (US Dollar), or both (Leu), respectively. The transmission did not succeed after contact with the contaminated Euro banknotes, in any of 3 subjects (Table 3). Transmission of the Romanian Leu (*E. coli* and MSSA) and of the US Dollar (MSSA) was successful, with the amount of colony forming units slightly higher after contact with the Leu.

**Table 3:** **Transmission of *E.coli *and/or *S. aureus *from banknotes to volunteers**

**Currency**	**Micro-organism**	**Culture results**		
		**Person I**	**Person II**	**Person III**
**Euro**	***E. coli***	Ø	Ø	Ø
**US Dollar**	***S. aureus***	**+**	**+**	**+**
**Romanian Leu (RON)**	***E. coli***	**++**	**++**	**++**
	***S. aureus***	**++**	**++**	**++**

++ = multiple segments, + = single colonies, Ø = no growth.

## Discussion

Banknote paper is manufactured from cotton fibre, which gives the paper its strength, durability and distinctive feel [10]. The cotton is sometimes mixed with linen, abaca, or other textile fibres. Unlike most printing and writing paper, banknote paper is infused with polyvinyl alcohol or gelatin to give it extra strength. Polymer (or plastic) banknotes were developed to improve durability and prevent counterfeiting through incorporated security features, such as optically variable devices that are extremely difficult to reproduce [11]. Romania, Israel, Malaysia and some other countries have adopted these polymer-containing banknotes [12]. Probably, the content of banknotes is also a factor that affects the survival of bacteria on the banknotes.

Our experiments showed that the polymer structure of the Romanian Leu banknote allows growth and transmission of multi-drug resistant pathogens. This, in theory, could contribute to the transmission of microorganisms within the Romanian community. Countries using polymer-based banknotes should take this into consideration, especially, if a currency is not exclusively used within one country, such as the Euro and US Dollar. Despite prolonged survival of *E. coli* on the Euro (up to 6 hours), transmission to the volunteers did not succeed. In contrast, US and Canadian Dollar showed significant and prolonged carriage of MRSA. *S. aureus* was transmissible from the US Dollar and thus indicates, that banknotes may play a role in the transmission of MRSA within the community. The Croatian Kuna was found to, unexpectedly, not allow growth of any of the multi-drug resistant microorganisms tested. While we could not find further information on the content and make-up of this currency, it could be interesting for other countries who want to eliminate banknotes as a source of bacterial transmission. Gram-positive and –negative microorganisms, including staphylococci, bacilli, and various enterobacteriaceae, have been found on banknotes in multiple countries, such as in Iran [8] and Turkey [12].

Money is frequently touched during daily life. The observed differences between the various currencies were unexpected and were not based on different climate conditions or hygiene levels, since all experiments were performed at the same conditions, using previously sterilized banknotes. While further studies would be needed to establish transmission of multi-drug resistant microorganisms through contact with money, or experiments show that this may be a potential pathway, especially in countries that use polymer-based banknotes.

## Competing interest

The authors declare that they have no competing interests.

## Authors’ contribution

HG and AV contributed to the conception and design, HB and TAV to the analysis of the data. All authors (HG, TAV, AV) contributed to drafting the manuscript or revising it and all authors approved the final manuscript all authors.
